# Feasibility and clinical usefulness of deep learning-accelerated MRI for acute painful fracture patients wearing a splint: A prospective comparative study

**DOI:** 10.1371/journal.pone.0287903

**Published:** 2023-06-28

**Authors:** Seunghyeon Roh, Jae In Park, Gun Young Kim, Hye Jin Yoo, Dominik Nickel, Gregor Koerzdoerfer, JaeKon Sung, Jiseon Oh, Hee Dong Chae, Sung Hwan Hong, Ja-Young Choi

**Affiliations:** 1 Department of Radiology, Seoul National University Hospital, Seoul, Republic of Korea; 2 Department of Radiology, Seoul National University College of Medicine, Seoul, Republic of Korea; 3 Siemens Healthcare GmbH, Erlangen, Germany; 4 Siemens Healthineers Ltd, Seoul, Republic of Korea; Universitair Kinderziekenhuis Koningin Fabiola: Hopital Universitaire des Enfants Reine Fabiola, BELGIUM

## Abstract

**Objective:**

To evaluate the feasibility and clinical usefulness of deep learning (DL)-accelerated turbo spin echo (TSE_DL_) sequences relative to standard TSE sequences (TSE_S_) for acute radius fracture patients wearing a splint.

**Methods:**

This prospective consecutive study investigated 50 patients’ preoperative wrist MRI scans acquired between July 2021 and January 2022. Examinations were performed at 3 Tesla MRI with body array coils due to the wrist splint. Besides TSE_S_ obtained according to the routine protocol, TSE_DL_ sequences for axial T2-, coronal T1-, and coronal PD-weighted TSE sequences were scanned for comparison. For quantitative assessment, the relative signal-to-noise ratio (rSNR), the relative contrast-to-noise ratio (rCNR), and the relative contrast ratio (rCR) were measured. For qualitative assessment, all images were assessed by two independent musculoskeletal radiologists in terms of perceived SNR, image contrast, image sharpness, artifacts disturbing evaluation, overall image quality and diagnostic confidence for injuries using a four- or five-point Likert scale.

**Results:**

The scan time was shortened approximately by a factor of two for TSE_DL_ compared to TSE_S_. TSE_DL_ images showed significantly better rSNR, rCNR, and rCR values for all sequences, and scored significantly better in terms of both image quality and diagnostic confidence for both readers than TSE_S_ images (all *p* < .05). Interrater reliabilities were in almost perfect agreement.

**Conclusion:**

The DL-accelerated technique proved to be very helpful not only to reduce scan time but also to improve image quality for acute painful fracture patients wearing a splint despite using body array coils instead of a wrist-specific coil. Based on our study, the DL-accelerated technique can be very useful for MRI of any part of the extremities in trauma settings just with body array coils.

## Introduction

The distal radius is one of the most common sites of fracture, and the incidence of distal radius fracture is increasing among people in all age groups. It accounts for approximately 18% of fractures in patients 65 years and older, and it hinders one’s ability to perform daily activities, such as preparing meals and performing housekeeping duties [1–3].

Furthermore, associated soft tissue injuries (e.g., intrinsic scapholunate and lunotriquetral ligament, triangular fibrocartilage with or without concomitant distal radioulnar joint instability) are reported with high incidence [3]; MRI evaluation is crucial for the detection of these soft tissue injuries [4]. Additionally, MRI is used to identify potential radiographically occult fractures, such as associated occult scaphoid fractures [5]. More than 60% of patients diagnosed with distal radius fracture undergo surgical correction [6]. In the case of acute wrist fracture patients in need of surgical correction, preoperative MRI scans might have an important role in the evaluation of associated soft tissue injury and surgical planning.

When patients with acute distal radius fracture need to undergo preoperative MRI, they are prone to suboptimal MRI quality for the following reasons. These patients tend to undergo manual reduction and splinting as soon as possible after diagnosis to prevent further injury. However, the splints are not easily detachable, and with the wrist splint on, wrist-specific coils are not suitable for MRI scans, causing impaired image quality and subsequent inaccurate diagnosis for soft tissue injuries. Additionally, patients are in such pain that it is difficult for them to remain still for the long time required for an MRI scan, which makes them susceptible to motion artifacts. Over the years, various attempts have been made to reduce the scan time, such as parallel imaging [7–9] and compressed sensing [10–12]. More recently, deep learning (DL)-based reconstruction and acceleration techniques have been developed. With concurrent denoising, DL-based reconstruction and acceleration techniques have achieved some promising results in reducing scan time and improving image quality simultaneously for various body parts, including the prostate [13], pituitary gland [14], liver [15], pelvis [16], shoulder and hip joints [17]. A preemptive study about the feasibility of implementing DL reconstruction in musculoskeletal turbo spin echo (TSE) imaging has been conducted with positive results [18]. To the best of our knowledge, no previous studies have applied deep learning-accelerated MRI in a painful trauma clinical setting. It was hypothesized in this study that with this DL approach, high image quality wrist MRI scans can be acquired and a reduction in scan time can be achieved simultaneously, when utilizing body array coils instead of a wrist-specific coil.

The purpose of this study was to evaluate the feasibility and clinical usefulness of deep learning-accelerated turbo spin echo (TSE_DL_) sequences relative to standard TSE (TSE_S_) sequences for acute distal radius fracture patients wearing a splint on the wrist.

## Materials and methods

This prospective study was approved by the institutional review board of Seoul National University Hospital, and written informed consent was obtained from all subjects before inclusion in the study (IRB No. H-2105-079-1218).

### Study population

Fifty-three consecutive consenting patients with acute distal radius fracture diagnosed based on wrist CT scan findings and who were scheduled for preoperative wrist MRI were prospectively included in this study between July 2021 and January 2022. Three patients were excluded for the following reasons: One had a scaphoid fracture with screw fixation in the affected wrist. Another two were in so much pain that they had to end the scanning process as fast as possible. Finally, 50 patients (41 female and 9 male patients; mean age ± SD, 64.68 ± 10.83 years; age range 31–86 years) were included in this study.

### Image acquisition

**Fig 1:**
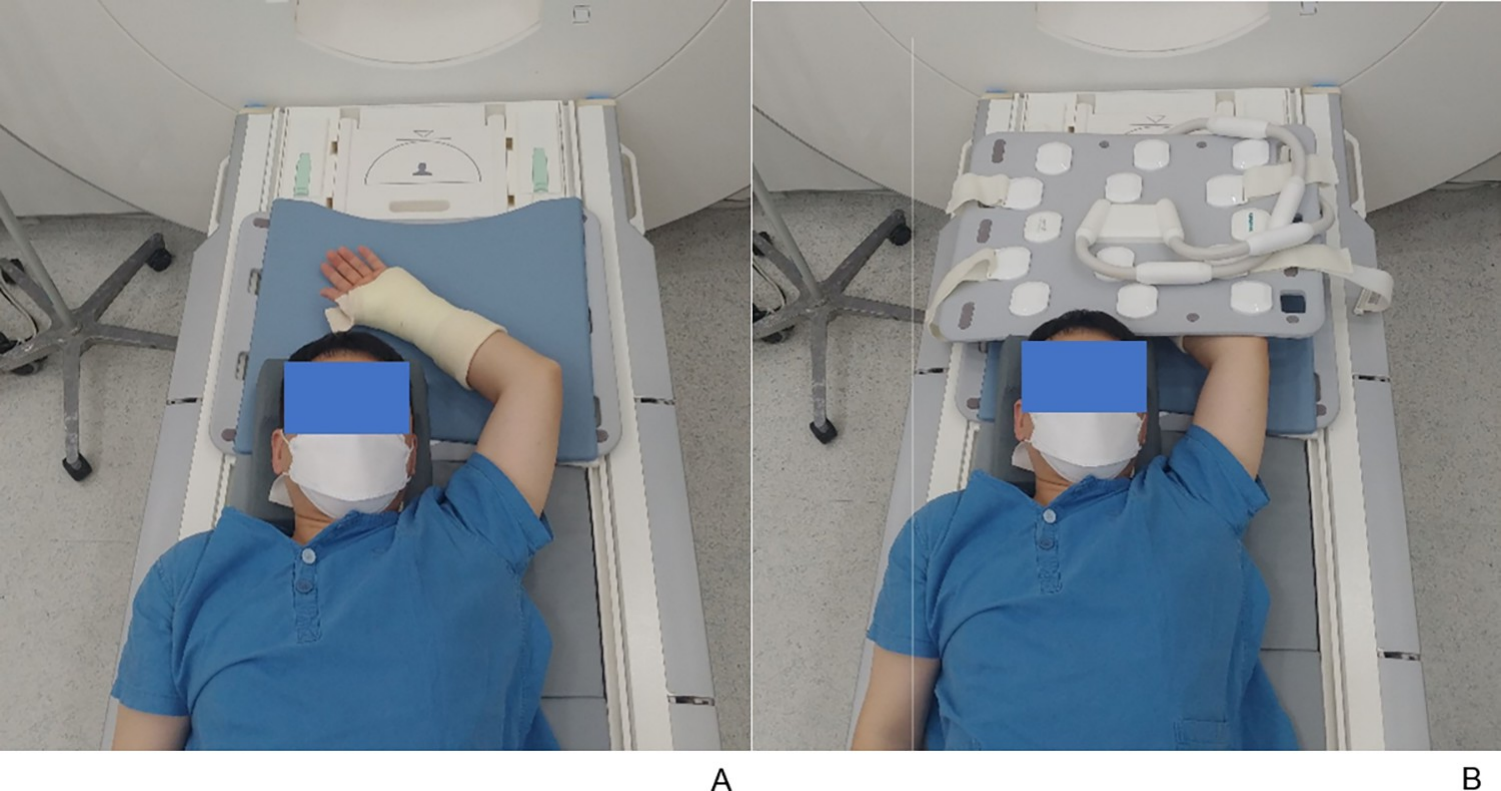
Patient’s scan position with a pair of 30-channel body array coils. (A) The patient with sugar-tong splint immobilization in the supine position, (B) and a pair of 30-channel body array coils in position.

All MRI examinations were performed using a 3 Tesla MRI scanner (MAGNETOM Skyra^fit^, Siemens Healthcare) with a pair of 30-channel body array coils (Body 30, Siemens Healthcare) instead of a wrist-specific coil. All study participating patients who underwent sugar-tong splint immobilization for distal radius fracture (anteroposterior compression after partial closure reduction via traction and release to prevent joint dislocation; the distal end of the splint was not to cross the distal ends of the metacarpal bones) were scanned in supine position with the hand above the head (Fig 1). First, TSE_S_ sequences were obtained according to the routine MRI protocol for distal radius fractures in our institute, which was as follows: axial T2-weighted TSE_S_ sequence with fat suppression (FS), axial T1-weighted TSE_S_ sequence, coronal T1-weighted TSE_S_ sequences, coronal T2-weighted and PD-weighted TSE_S_ sequences with FS, and sagittal T2-weighted TSE_S_ sequences. In addition, axial T2-weighted TSE_DL_ sequence with FS, coronal T1-weighted TSE_DL_ sequence, and coronal PD-weighted TSE_DL_ sequence with FS were acquired for comparison. The acquisition parameters for each sequence are summarized in Table 1.

**Table 1:** Summary of sequence acquisition parameters.

	T1 COR TSE_S_	T1 COR TSE_DL_	PD COR TSE_S_ FS	PD COR TSE_DL_ FS	T2 AX TSE_S_ FS	T2 AX TSE_DL_ FS
FOV (mm)	120×120	120×120	120×120	120×120	100×100	100×100
Acq Voxel (mm)	0.33×0.27	0.33×0.27	0.39×0.31	0.39×0.31	0.32×0.26	0.32×0.26
Recon Matrix	448×352	448×352	384×311	384×311	384×311	384×311
Recon Voxel (mm)	0.27×0.27	0.27×0.27	0.31×0.31	0.31×0.31	0.26×0.26	0.26×0.26
NEX	2	1	2	1	2	1
TR (ms)	672	672	2930	2930	2500	2500
TE (ms)	15	15	27	27	57	57
Phase encoding	F-H	F-H	F-H	F-H	A-P	A-P
Flip angle (°)	160	160	160	160	160	160
NO. of Slice	24	24	24	24	26	26
PAT	4	4	3	3	3	3
Bandwidth (Hz)	149	149	148	148	148	148
Slice Thickness (mm)	2/0	2/0	2/0	2/0	3/0	3/0
Scan Time (min:sec)	3:31	1:46	4:24	2:15	4:00	2:05

Note—Note that the scan time for each TSE_DL_ is approximately half of the corresponding TSE_S_.

TSE_S_: standard turbo spin echo, TSE_DL_: accelerated deep learning-based turbo spin echo, T1 COR: coronal T1-weighted image, PD COR: coronal proton density-weighted image, T2 AX: axial T2-weighted image, FS: with fat saturation, FOV: field of view, F-H: foot-head, A-P: anterior-posterior, NEX: number of excitation, TR: time of repetition, TE: time of echo, PAT: Parallel Acquisition Technique

The prototypical TSE_DL_ sequence employs a deep-learning reconstruction which is designed for improving the signal-to-noise ratio of acquisitions with higher accelerations. It comprises an unrolled variational network [19] and we refer to references [13, 18] for more details on the technical implementation used in this study.

### Quantitative image analysis

**Fig 2:**
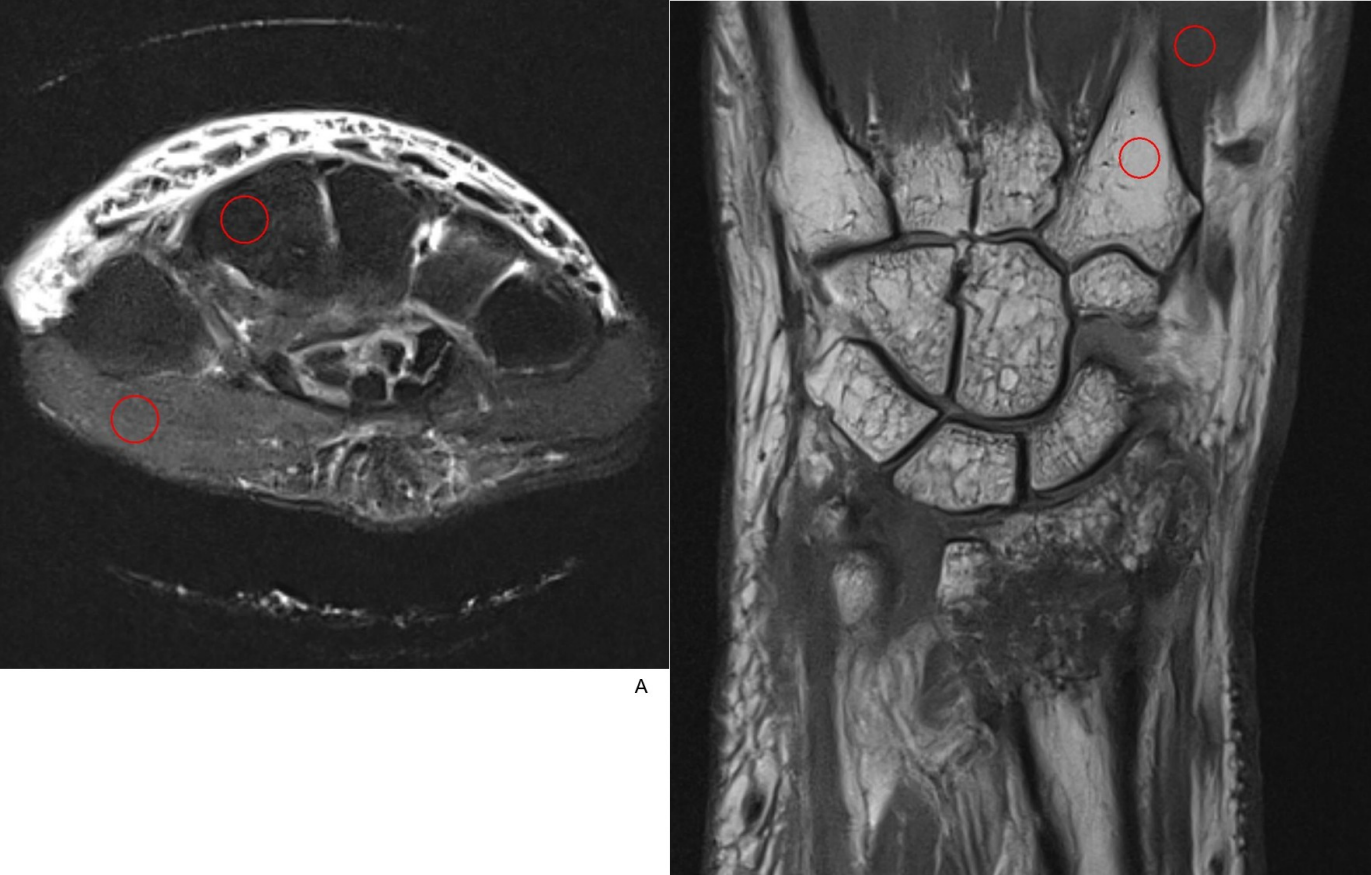
ROI placement. (A) A 5 mm circular ROI was placed at the thenar muscle and the 2nd metacarpal base in axial fat-suppressed image. (B) For coronal images, the 1^st^ interosseous muscle and 2nd metacarpal base of the same image plane were selected.

For objective comparison of image quality, the relative signal-to-noise ratio (rSNR) for bone and muscle, relative contrast-to-noise ratio (rCNR), and relative contrast ratio (rCR) between bone and muscle were measured on TSE_DL_ and TSE_S_ images by a 2^nd^ year radiology resident (S.H.R). For the corresponding TSE_DL_ and TSE_S_ images of each sequence, the same sized circular ROIs with a diameter of 5 mm were placed in the same location. For axial images, the thenar muscle and 2^nd^ metacarpal base of the same plane were selected for muscle and bone analysis measurements (Fig 2A). For six patients whose 2^nd^ metacarpal base was not covered in the axial scan, the flexor digitorum muscle and distal radius or ulna of the same plane were selected instead. For coronal images, the 1^st^ interosseous muscle and 2^nd^ metacarpal base of the same image plane were selected (Fig 2B). Circular ROIs were placed in homogeneous areas away from other structures, such as vessels, edema or cysts. Regions affected with partial volume averaging were avoided as well. We measured signal intensity (SI) values and standard deviation (SD) within the ROI for comparison analysis. With those values, rSNR for bone and muscle, rCNR and rCR between bone and muscle were calculated using the following expressions [14, 17]:

$$SNR=\frac{SI}{SD}$$

$$CNR=\frac{\left|{SI}_{bone}-{SI}_{muscle}\right|}{mean({SD}_{bone},\;{SD}_{muscle})}$$

$$CR=\frac{\left|{SI}_{bone}-{SI}_{muscle}\right|}{mean({SI}_{bone},\;{SI}_{muscle})}$$

$${rSNR}_{DL}=\frac{{SNR}_{DL}}{max({SNR}_{DL},\;{SNR}_{S})}{,\;rSNR}_{S}=\frac{{SNR}_{S}}{max({SNR}_{DL},\;{SNR}_{S})}$$

$${rCNR}_{DL}=\frac{{CNR}_{DL}}{max({CNR}_{DL},\;{CNR}_{S})}{,\;rCNR}_{S}=\frac{{CNR}_{S}}{max({CNR}_{DL},\;{CNR}_{S})}$$

$${rCR}_{DL}=\frac{{CR}_{DL}}{max({CR}_{DL},\;{CR}_{S})},\;{rCR}_{S}=\frac{{CR}_{S}}{max({CR}_{DL},\;{CR}_{S})}$$

### Qualitative image analysis

**Fig 3:**
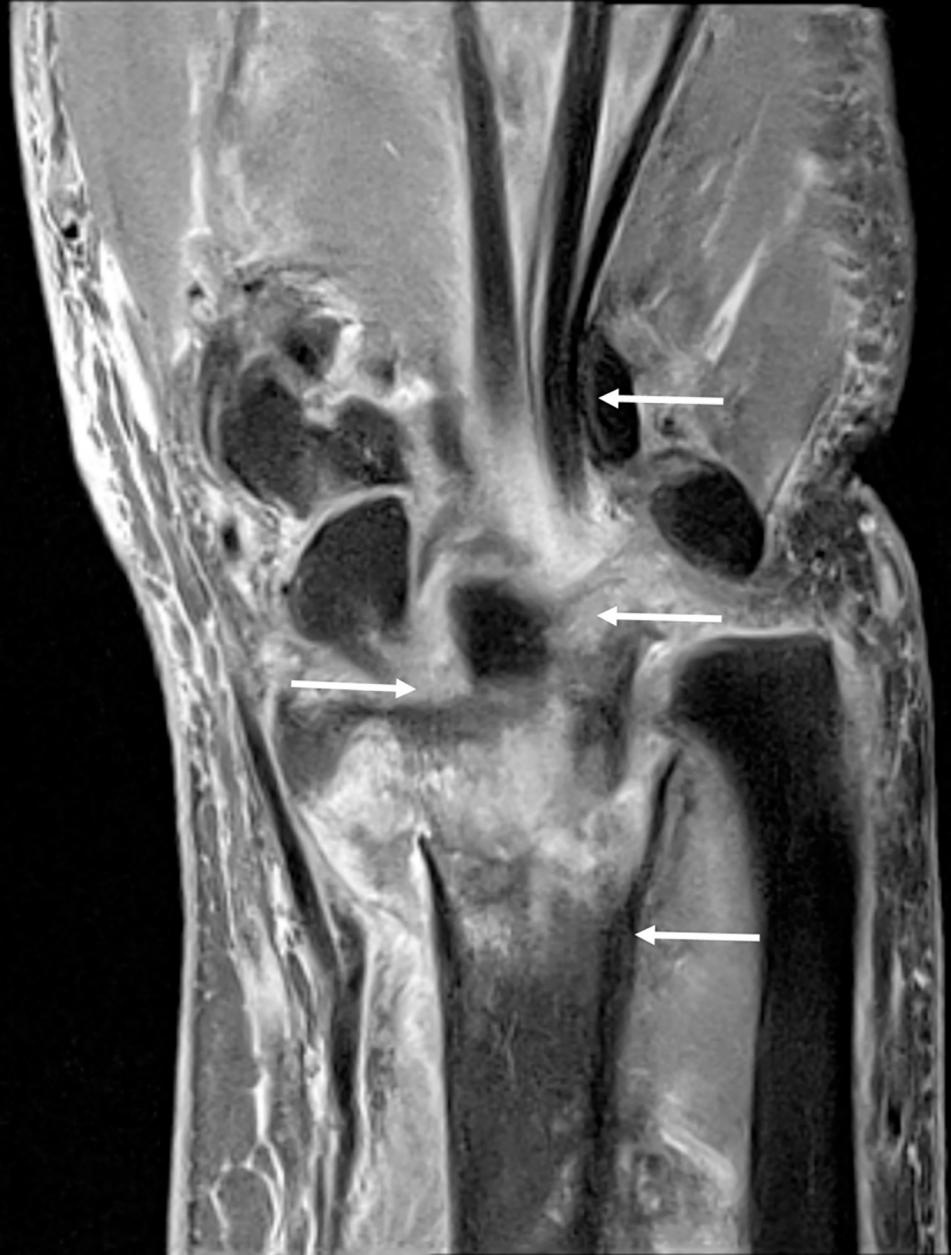
Grid artifacts. An 80-year-old female with a distal radius fracture. Coronal PD-weighted TSE_DL_ image with fat suppression showed grid artifact (arrows), which was defined as a pattern of lines that cross each other to form squares on the image.

All images were independently evaluated by two independent board-certified musculoskeletal radiologists (H.J.Y. with 17 years of experience; J.Y.C., with 23 years of experience). For each case, two image sets of TSE_DL_ and TSE_S_ sequences were anonymized and distributed in random order. The readers were blinded to the clinical information, radiological report, and scan parameters. For each image set, image quality parameters, including the perceived signal-to-noise ratio, image contrast, image sharpness, artifacts (motion, grid) disturbing evaluation, and overall image quality, were evaluated using a 4-point Likert scale (Table 2). Grid artifact was defined as a pattern of coarse lines that cross each other to form squares on the images (Fig 3). In addition, diagnostic confidence levels were assessed in terms of the presence of a distal radius fracture, ulnar styloid fracture/bone contusion, and triangular fibrocartilaginous complex (TFCC) injury. Diagnostic confidence for each abnormality was measured based on a 5-point Likert scale. Detailed information on the scale used for qualitative evaluation of image quality and diagnostic confidence is provided in Table 2.

**Table 2:** Scoring scale for various parameters in qualitative image analysis.

**Imaging quality parameter**	**Scoring system**
Perceived signal-to-noise ratio	1: Too noisy for interpretation 2: Noise has an adverse effect on interpretation. 3: Perceivable noise, but no adverse effect for interpretation. 4: Neglectable or no noticeable noise.
Image contrast	1: One of the structures is not separated. 2: All of the structures are separated, but the contrast is weak. 3: All of the structures are mostly separated. 4: All of the structures are clearly separated.
Image sharpness	1: Poor 2: Fair (structure fully visible but substantial blurring of borders) 3: Good (structure fully visible but slight blurring of borders) 4: Neglectable or no noticeable artifacts
Artifacts disturbing evaluation	1: Excessive artifacts with distortion of images 2: Hampered readability due to artifacts 3: Slight artifacts without limited readability 4: Neglectable or no noticeable artifacts
Overall image quality	1: Poor 2: Fair 3: Good 4: Excellent.
**Diagnostic confidence**	**Scoring system**
Distal radius fracture	1: Certainly absent 2: Probably absent 3: Uncertain 4: Probably present (fracture lines and bone marrow edema visualized, but slight blurred) 5: Certainly present (excellent conspicuity of the fracture lines and surrounding bone marrow margin)
Ulnar styloid fracture/bone contusion	1: Certainly absent 2: Probably absent 3: Uncertain 4: Probably present—fracture lines and bone marrow edema visualized, but slight blurred 5: Certainly present—excellent conspicuity of the fracture lines and surrounding bone marrow margin
Triangular fibrocartilaginous complex (TFCC) injury	1: Certainly absent 2: Probably absent 3: Uncertain 4: Probably present—tear (thinning/discontinuity) or intrasubstance signal abnormality visualized, but slight blurring 5: Certainly present—excellent conspicuity of the tear (thinning/discontinuity) or intrasubstance signal abnormality

Note—Two independent board-certified musculoskeletal radiologists evaluated image quality and diagnostic confidence using a 4- or 5-point Likert scale for qualitative image analysis.

### Statistical analysis

For comparison of the rSNR, rCNR, and rCR between TSE_DL_ and TSE_S_ sequences, the paired t test was used. For qualitative image analysis and diagnostic confidence, the Wilcoxon signed rank test was utilized. A *p value* of less than .05 was considered to indicate a significant difference. All statistical analyses were performed by using commercially dedicated software (IBM SPSS Statistics 27 software for Windows; IBM). The levels of interrater agreement were evaluated using Gwet’s agreement coefficient (AC) [20] due to the skewed marginal distribution of qualitative scores [21]. Gwet’s ACs were calculated using STATA/MP 17 for Windows; StataCorp LLC [22]. Interrater reliability was categorized as poor (<0), slight (0–0.2), fair (0.21–0.4), moderate (0.41–0.6), substantial (0.61–0.8), or almost perfect agreement (0.8–1) [23].

## Results

The total scan time for axial T2-, coronal T1-, and coronal PD-weighted TSE sequences was 11 minutes and 55 seconds for TSE_S,_ and 6 minutes and 6 seconds for TSE_DL_ (Table 1).

### Quantitative image quality assessment

**Fig 4:**
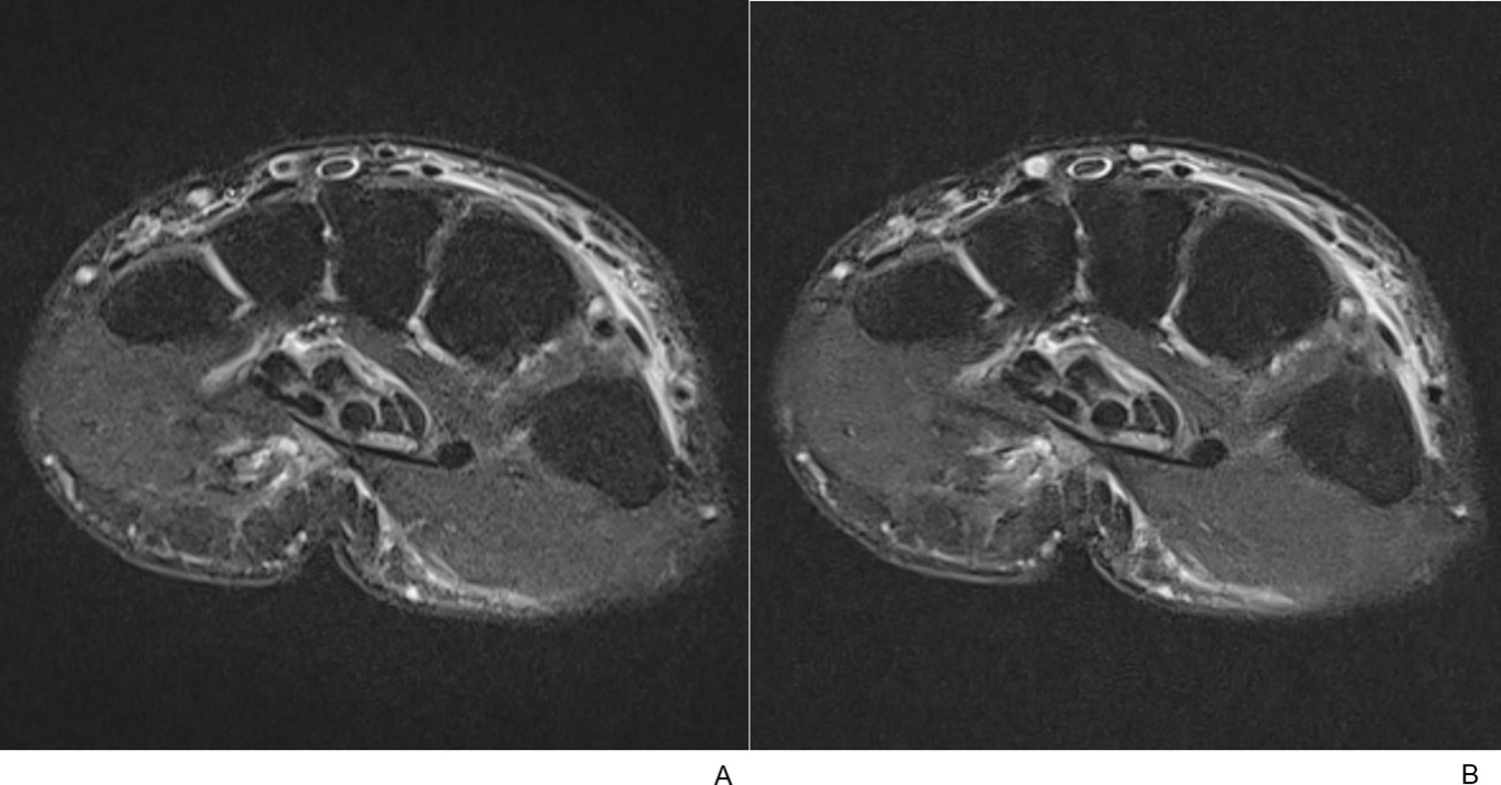
A 57-year-old female with a distal radius fracture. (A) Axial T2-weighted TSE_S_ image with fat suppression and (B) the corresponding TSE_DL_ image. TSE_DL_ images showed superior rSNR, rCNR and rCR compared to TSE_S_ images.

**Fig 5:**
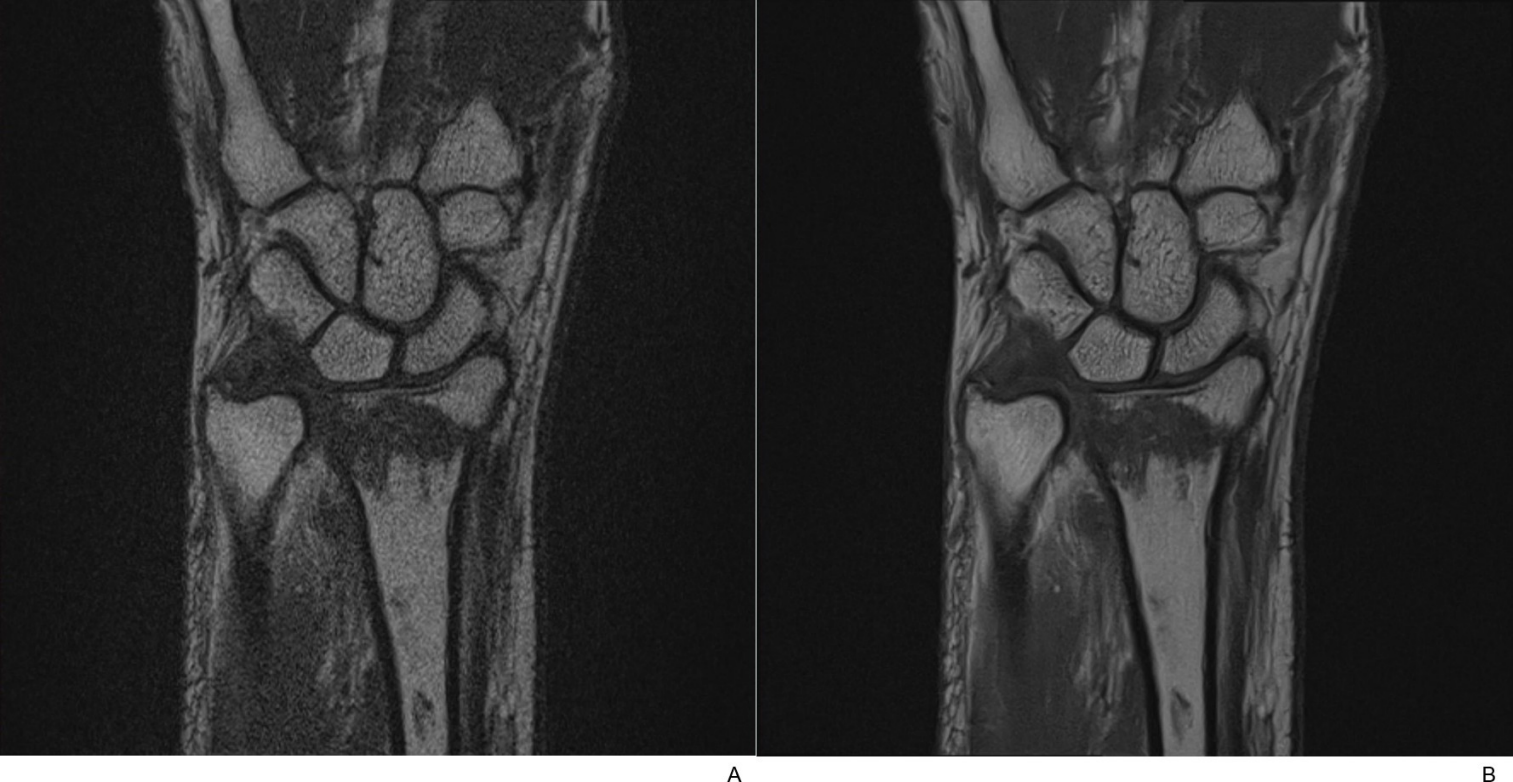
A 57-year-old female with a distal radius fracture. (A) Coronal T1-weighted TSE_S_ image and (B) the corresponding TSE_DL_ image. TSE_DL_ images showed superior rSNR, rCNR and rCR compared to TSE_S_ images.

**Fig 6:**
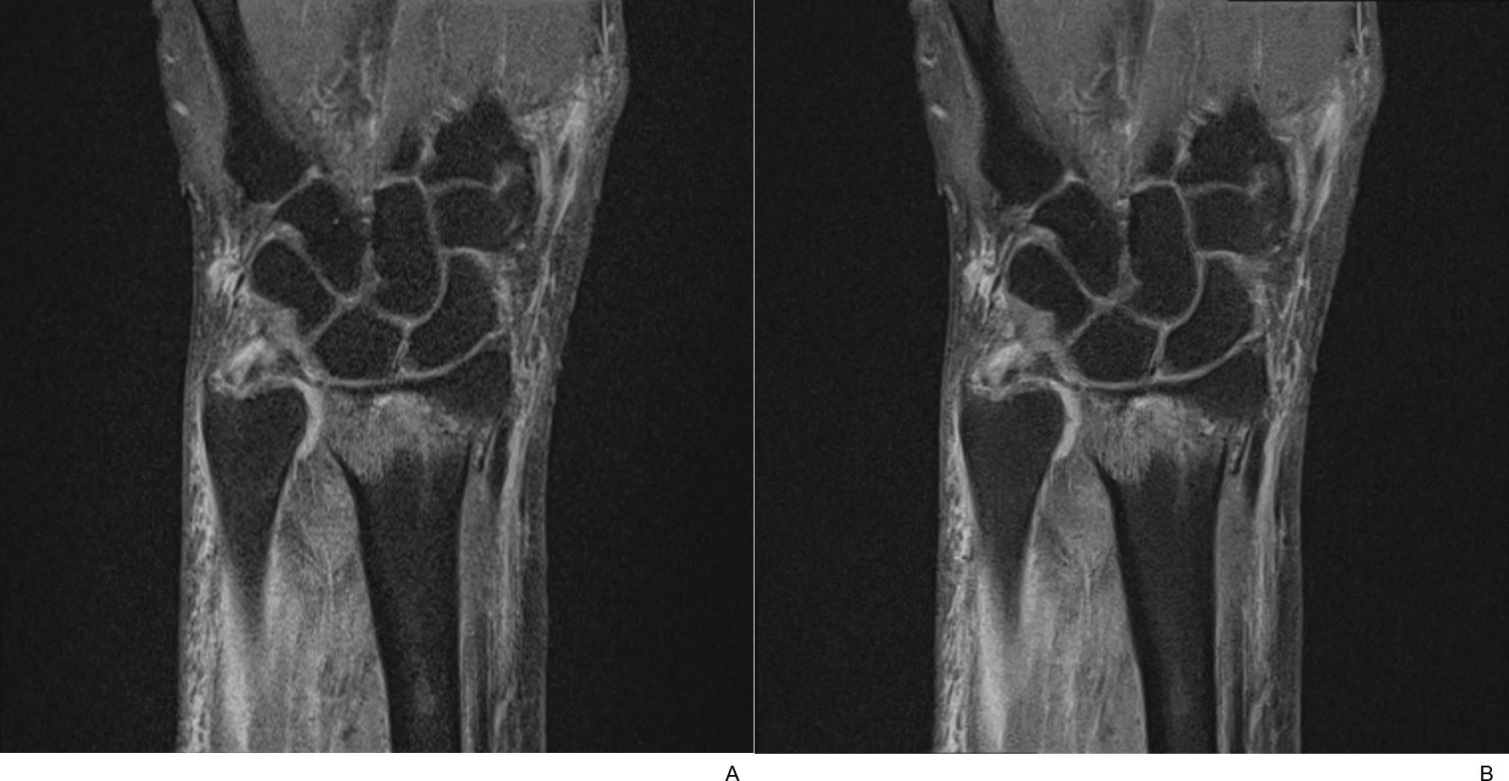
A 57-year-old female with a distal radius fracture. (A) PD-weighted TSE_S_ image with fat suppression and (B) the corresponding TSE_DL_ image. TSE_DL_ images showed superior rSNR, rCNR and rCR compared to TSE_S_ images.

TSE_DL_ images showed significantly better (*p* < .05) bone rSNR and muscle rSNR for all sequences than TSE_S_ images; axial T2-, coronal T1-, and coronal PD-weighted TSE (Table 3). TSE_DL_ images showed significantly better (*p* < .05) rCNR and rCR between bone and muscle for all sequences as well (Table 3). See Figs 4–6 for example images.

**Table 3:** Quantitative assessment of image quality between TSE_DL_ and TSE_S_ sequences.

		TSE_DL_	TSE_S_	*p* value
**AxialT2WIFS**	**rSNR (bone)**	0.99 ± 0.02 (0.96–1.03)	0.84 ± 0.10 (0.80–0.87)	< 0.001*
	**rSNR (muscle)**	1.00 ± 0.02 (0.96–1.03)	0.82 ± 0.10 (0.78–0.85)	<0.001*
	**rCNR**	1.00 ± 0.00 (0.97–1.03)	0.81 ± 0.09 (0.78–0.83)	<0.001*
	**rCR**	0.99 ± 0.03 (0.97–1.00)	0.97 ± 0.04 (0.95–0.98)	0.027*
**CoronalT1WI**	**rSNR (bone)**	1.00 ± 0.00 (0.96–1.04)	0.71 ± 0.16 (0.66–0.75)	<0.001*
	**rSNR (muscle)**	1.00 ± 0.00 (0.97–1.03)	0.48 ± 0.10 (0.45–0.50)	<0.001*
	**rCNR**	1.00 ± 0.00 (0.96–1.04)	0.63 ± 0.13 (0.59–0.66)	<0.001*
	**rCR**	1.00 ± 0.00 (0.97–1.03)	0.75 ± 0.09 (0.72–0.78)	<0.001*
**CoronalPDWIFS**	**rSNR (bone)**	0.99 ± 0.07 (0.94–1.03)	0.85 ± 0.11 (0.80–0.89)	<0.001*
	**rSNR (muscle)**	0.99 ± 0.04 (0.95–1.02)	0.86 ± 0.11 (0.82–0.89)	<0.001*
	**rCNR**	0.99 ± 0.03 (0.96–1.02)	0.82 ± 0.09 (0.79–0.86)	<0.001*
	**rCR**	0.98 ± 0.04 (0.96–1.01)	0.96 ± 0.06 (0.94–0.98)	0.043*

Note—Comparison of rSNR, rCNR and rCR between each corresponding TSE_DL_ and TSE_S_ sequence. Values are presented as the mean ± standard deviation (95% confidence interval of paired t test).

TSE_S_: standard turbo spin echo, TSE_DL_: accelerated deep learning-based turbo spin echo, rSNR: relative signal-to-noise ratio, rCNR: relative contrast-to-noise ratio, rCR: relative contrast ratio

*: statistically significant (*p* < .05)

### Qualitative evaluation of image quality and diagnostic confidence

**Fig 7:**
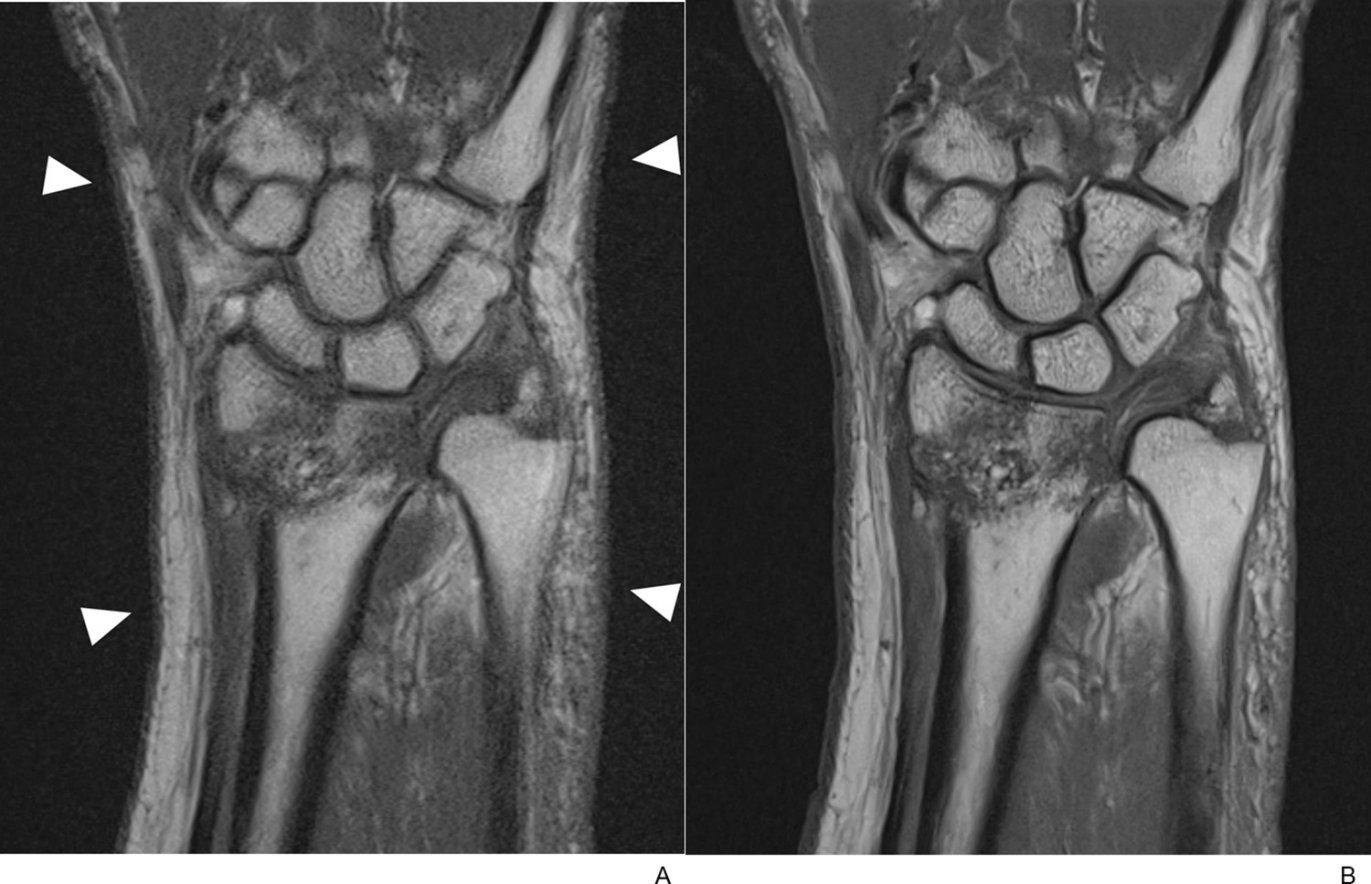
Motion artifacts. (A) Coronal T1-weighted TSE_S_ image of a 70-year-old female patient revealed severe margin blurring of the bone and joint by motion artifact (arrowheads). (B) In contrast, sharp-edged anatomic details were noted without motion artifacts in the corresponding TSE_DL_ image.

**Fig 8:**
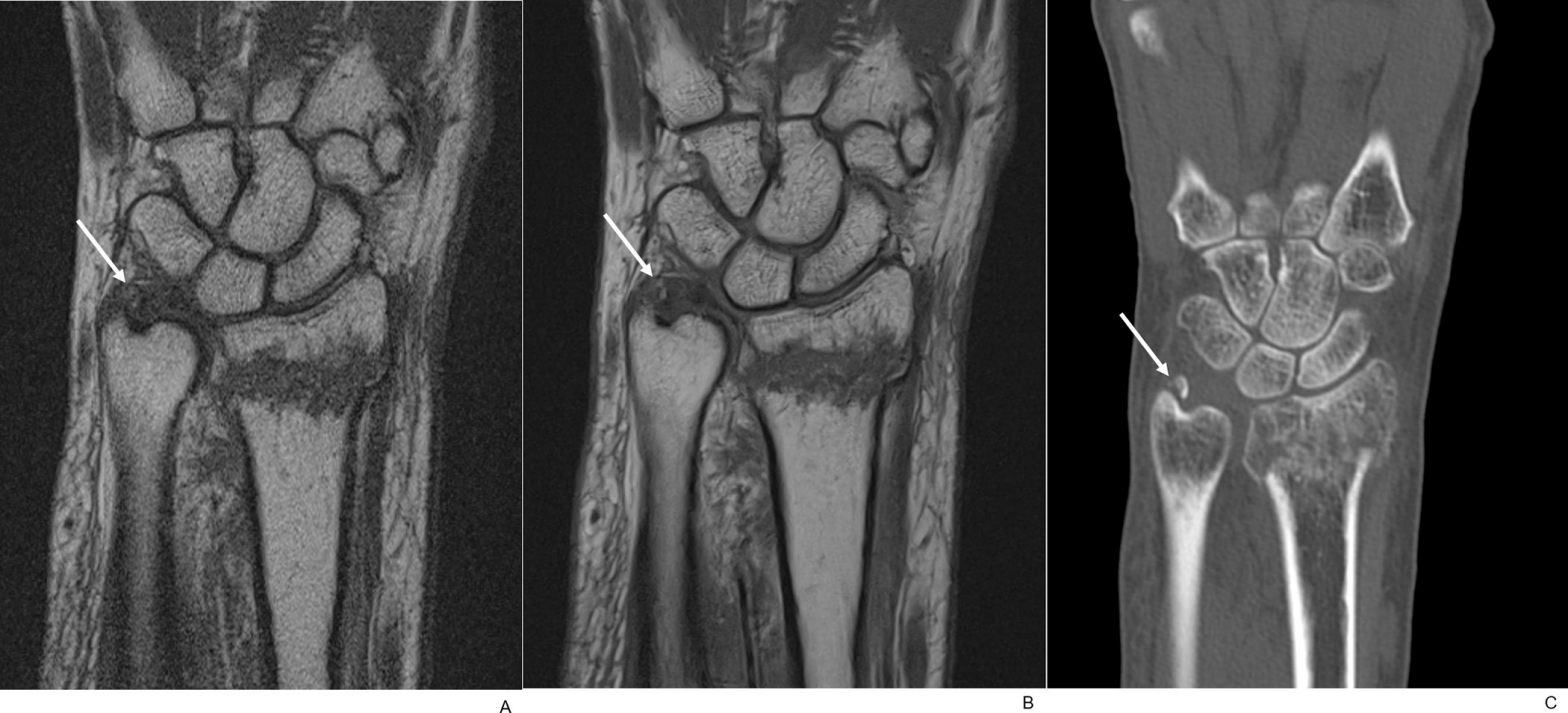
Associated ulnar styloid process fracture. (A) Coronal T1-weighted TSE_S_ image of a 60-year-old female with a distal radius fracture demonstrated suspected fragmentation of the ulnar styloid process with indistinct cortices. (B) However, a small bony fragment with distinct cortices and internal fatty marrow was well delineated on the corresponding TSE_DL_ image. (C) Coronal CT image clearly showed ulnar styloid process fracture with bone fragments.

**Fig 9:**
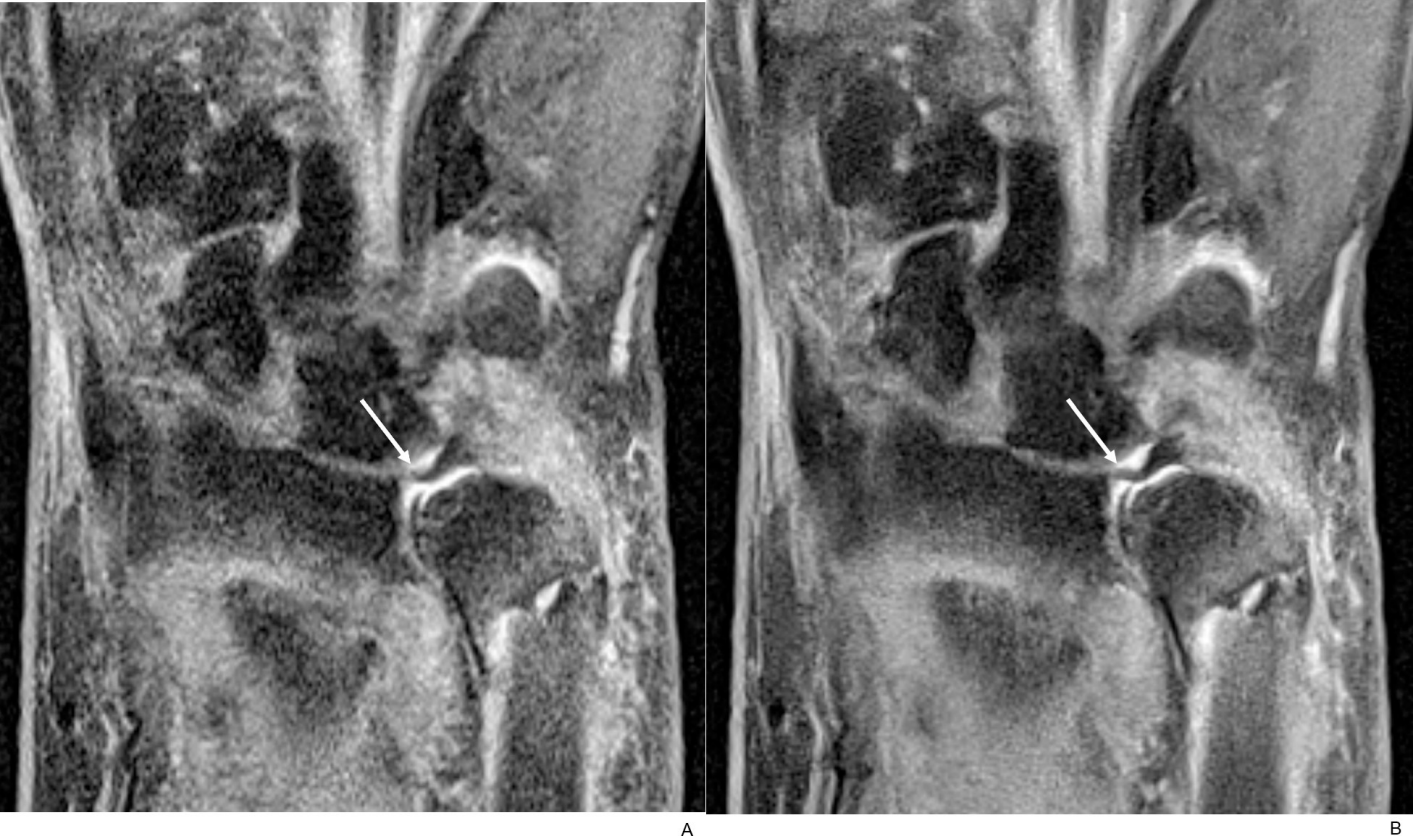
Associated triangular fibrocartilage complex injury. (A) On coronal fat-suppressed PD-weighted TSE_S_ image of an 86-year-old female with distal radius and ulnar fractures, wavy radial attachment (arrow) of the disc was seen with blurred margin and internal signal alteration. (B) In contrast, the TSE_DL_ image showed a sharp margin of the disc with localized signal alteration of the radial attachment (arrow). Nearby lunate articular cartilage and ulnar cortical fragments were clearly delineated as well.

TSE_DL_ sequences demonstrated significantly better image quality than TSE_S_ sequences for both readers (*p* < .05) in terms of perceived SNR, image contrast, image sharpness, motion artifacts and overall image quality (Table 4, Fig 7). Regarding diagnostic confidence, TSE_DL_ sequences showed significantly higher confidence levels for all distal radius fractures, ulnar styloid fractures (Fig 8), and TFCC injuries (Fig 9) (*p* < .001 in all) for both readers except one reader’s TFCC injury evaluation, which was due to both TSE_DL_ sequences and TSE_S_ sequences scoring the highest point for all patients. Interrater reliabilities showed almost perfect agreement for qualitative evaluation of image quality and diagnostic confidence except in the following cases: distal radius fracture and ulnar styloid fracture/bone contusion diagnostic confidence for TSE_S_ sequences (moderate) and image sharpness for TSE_S_ sequence (fair) (Table 4).

**Table 4:** Qualitative assessment of image quality between TSE_DL_ and TSE_S_ sequences.

	TSE_DL_	Gwet AC	TSE_S_	Gwet AC	*p* value
Perceived SNR					
Reader 1	4 (4, 4)	0.73 (0.55, 0.91)	3 (3, 3)	0.94 (0.86, 1.00)	<0.001**
Reader 2	4 (4, 4)		3 (3, 3)		<0.001**
Image contrast					
Reader 1	4 (4, 4)	0.094 (0.86, 1.00)	4 (4, 4)	0.073 (0.54, 0.91)	0.046*
Reader 2	4 (4, 4)		4 (4, 4)		0.007*
Image sharpness					
Reader 1	4 (4, 4)	0.76 (0.59, 0.92)	3 (3, 3)	0.26 (-0.05, 0.57)	<0.001**
Reader 2	4 (4, 4)		3 (3, 4)		<0.001**
Artifacts (grid)					
Reader 1	4 (4, 4)	0.74 (0.56, 0.92)	4 (4, 4)	0.96 (0.90, 1.00)	0.002*
Reader 2	4 (4, 4)		4 (4, 4)		0.008*
Artifacts (motion)					
Reader 1	4 (4, 4)	0.98 (0.94, 1.00)	4 (4, 4)	0.82 (0.69, 0.95)	0.014*
Reader 2	4 (4, 4)		4 (4, 4)		0.033*
Overall image quality					
Reader 1	4 (4, 4)	0.91 (0.82, 1.00)	3 (3, 3)	0.62 (0.40, 0.84)	<0.001**
Reader 2	4 (4, 4)		3 (3, 3.75)		<0.001**
Distal radius fracture					
Reader 1	5 (5, 5)	PF	4 (4, 5)	0.58 (0.35, 0.82)	<0.001**
Reader 2	5 (5, 5)		4 (4, 4)		<0.001**
Ulnar styloid fracture / Bone contusion					
Reader 1	5 (1.75, 5)	0.72 (0.57, 0.86)	4 (1, 4)	0.58 (0.40, 0.75)	<0.001**
Reader 2	5 (1, 5)		4 (1, 4)		<0.001**
Triangular fibrocartilaginous complex (TFCC) injury					
Reader 1	5 (5, 5)	PF	5 (4, 5)	0.63 (0.45, 0.80)	<0.001**
Reader 2	5 (5, 5)		5 (5, 5)		1.000

Note—Comparison of image quality parameters between TSE_DL_ and TSE_S_ sequences evaluated by two independent board-certified musculoskeletal radiologists. Values are presented as the median (1^st^ quartile, 3^rd^ quartile).

TSE_S_: standard turbo spin echo, TSE_DL_: accelerated deep learning-based turbo spin echo, AC: agreement coefficient; PF: perfect match between Reader 1 and Reader 2 (Gwet’s AC not available)

**: statistically significant (*p* < .001)

## Discussion

In the current study, TSE_DL_ images showed significantly better rSNR, rCNR, and rCR than TSE_S_ images for all sequences for patients with acute painful distal radius fracture wearing a splint. For qualitative analysis, TSE_DL_ sequences were significantly better than TSE_S_ sequences in terms of both image qualities and diagnostic confidence.

It is inspiring that the TSE_DL_ sequences allow increased spatial resolution without image quality deterioration, while scan time is reduced by approximately half of that required for TSE_S_ sequences. It is difficult for patients with acute fracture to tolerate the long scan time due to pain even without motion. Therefore, we think that the longer it takes to acquire images, the more susceptible images are to motion artifacts (Fig 7). These artifacts may degrade image quality and subsequently lead to decreased diagnostic accuracy, thus incurring expensive costs for the patient and the institution due to potential exam failure or repeat exam. Our results showed that motion artifacts were less frequent in TSE_DL_ images than in TSE_S_ images. It goes without saying that reduced scan time itself is a considerable advantage for the patients and the institution in terms of convenience and cost-effectiveness.

Furthermore, in the present study, despite two unfavorable conditions for wrist MR scans, 1) using body array coils instead of a wrist-specific coil and 2) keeping the splint on at scan time (the culprit for off-center scanning or foreign body artifacts), TSE_DL_ images demonstrated excellent image quality. Based on our study, the TSE_DL_ technique can be very useful for MRI of any part of the extremities in trauma settings, such as fractures with splints or cast immobilization just with body array coils.

A disadvantage of TSE_DL_ images compared with TSE_S_ images was the minor ‘grid’ artifact that was sometimes observed (Fig 3). This minor byproduct hardly altered the diagnostic performance of the images, but it would be a candidate for future improvement.

There were several limitations in this study. First, although both quantitative and qualitative analysis generated congruent results, in favor of TSE_DL_ images over TSE_S_ images, there was no direct comparison between TSE_DL_ images and the hypothetical images with splint removal and wrist-specific coil application. However, it was difficult to remove the splint for a while during the MRI scan for comparison in daily practice, which might cause patient discomfort and failure of mechanical reduction. Second, this study investigated a vendor provided preset denoising level for DL-based reconstruction. However, appropriate levels of denoising could be further studied [14, 17], as too much denoising could impair the edge margins of distinct structures and impair image quality [13].

In conclusion, the deep learning-accelerated technique proved to be very helpful not only to reduce scan time but also to improve image quality simultaneously for patients with acute painful distal radius fracture wearing a splint on the wrist despite using body array coils instead of a wrist-specific coil. Based on our study, the deep-learning accelerated technique has potential for being applicable to MRI for any part of the extremities in trauma settings just with body array coils.

## Supporting information

S1 File (XLSX)

S2 File (XLSX)

S3 File (XLSX)
